# The RNA-Binding Protein, ZFP36L2, Influences Ovulation and Oocyte Maturation

**DOI:** 10.1371/journal.pone.0097324

**Published:** 2014-05-15

**Authors:** Christopher B. Ball, Karina F. Rodriguez, Deborah J. Stumpo, Fernando Ribeiro-Neto, Kenneth S. Korach, Perry J. Blackshear, Lutz Birnbaumer, Silvia B. V. Ramos

**Affiliations:** 1 Department of Obstetrics and Gynecology, Division of Reproductive Endocrinology and Infertility, University of North Carolina School of Medicine, Chapel Hill, North Carolina, United States of America; 2 Laboratory of Reproductive and Developmental Toxicology, National Institute of Environmental Health Sciences, National Institutes of Health, Department of Health and Human Services, Research Triangle Park, North Carolina, United States of America; 3 Laboratory of Signal Transduction, National Institute of Environmental Health Sciences, National Institutes of Health, Department of Health and Human Services, Research Triangle Park, North Carolina, United States of America; 4 Medicine and Biochemistry Departments, Duke University Medical Center, Durham, North Carolina, United States of America; 5 Laboratory of Neurobiology, National Institute of Environmental Health Sciences, National Institutes of Health, Department of Health and Human Services, Research Triangle Park, North Carolina, United States of America; Clermont-Ferrand Univ., France

## Abstract

ZFP36L2 protein destabilizes AU-rich element-containing transcripts and has been implicated in female fertility. In the C57BL/6NTac mouse, a mutation in *Zfp36l2* that results in the decreased expression of a form of ZFP36L2 in which the 29 N-terminal amino acid residues have been deleted, ΔN-ZFP36L2, leads to fertilized eggs that arrest at the two-cell stage. Interestingly, homozygous *ΔN-Zfp36l2* females in the C57BL/6NTac strain release 40% fewer eggs than the WT littermates (Ramos et al., 2004), suggesting an additional defect in ovulation and/or oocyte maturation. Curiously, the same *ΔN-Zfp36l2* mutation into the SV129 strain resulted in anovulation, prompting us to investigate a potential problem in ovulation and oocyte maturation. Remarkably, only 20% of *ΔN-Zfp36l2* oocytes in the 129S6/SvEvTac strain matured *ex vivo*, suggesting a defect on the oocyte meiotic maturation process. Treatment of *ΔN-Zfp36l2* oocytes with a PKA inhibitor partially rescued the meiotic arrested oocytes. Furthermore, cAMP levels were increased in *ΔN-Zfp36l2* oocytes, linking the cAMP/PKA pathway and *ΔN-Zfp36l2* with meiotic arrest. Since ovulation and oocyte maturation are both triggered by LHR signaling, the downstream pathway was investigated. Adenylyl cyclase activity was increased in *ΔN-Zfp36l2* ovaries only upon LH stimulation. Moreover, we discovered that ZFP36L2 interacts with the 3′UTR of LHR mRNA and that decreased expression levels of *Zfp36l2* correlates with higher levels of LHR mRNA in synchronized ovaries. Furthermore, overexpression of ZFP36L2 decreases the endogenous expression of LHR mRNA in a cell line. Therefore, we propose that lack of the physiological down regulation of LHR mRNA levels by ZFP36L2 in the ovaries is associated with anovulation and oocyte meiotic arrest.

## Introduction

Although some perceive infertility as a quality-of-life issue, it is in fact a disease with increasing public health concerns [1]. Infertility is a global health issue affecting 10–15% of the 1.5 billion women of reproductive age [2]. The majority of these women have no access to infertility treatment, and even in developed economies there are great inequalities in access to diagnosis and treatment [3]. Furthermore, when access to medical treatment is fully available, the basis of the infertility does not have a clear cause in 10–15% of the cases and is thus classified as ‘unexplained’, implying that the precise molecular basis of the infertility is unknown. Using knowledge obtained by studying a genetically engineered mouse model, we propose a new molecular basis for unexplained female infertility involving the ZFP36L2 RNA-binding protein's role in ovulation and oocyte maturation. Our results suggest that decreased ZFP36L2 expression can, conceivably, be the basis of some cases of unexplained female infertility in humans.

ZFP36L2 (Zinc Finger Protein 36 Like 2) is an RNA-binding protein that binds and destabilizes certain adenine/uridine rich element (ARE)-containing transcripts in cell transfection studies by promoting their deadenylation [4], [5]. ZFP36L2 is also known as TIS11D (Tetradecanoyl Phorbol Acetate (TPA)-Inducible Sequence 11D [6], ERF2 (Early Responsive Gene 2)[7], and BRF2 (Butyrate Response Factor 2) [8]. *In vivo*, ZFP36L2 is required for female fertility based on the phenotype observed in our previously genetically engineered mouse [9]. Homozygous mutant mice (-/-), *ΔN-Zfp36l2*, in which the *Zfp36l2* gene was disrupted at its first exon by NEO, resulted in a truncated protein, ΔN-ZFP36L2, expressed at lower levels and lacking 29 amino acids at its N-terminal [9]. This truncated protein is translated from a remaining transcript expressed at reduced levels, containing part of the single intron fused with exon 2. The lower expression levels of this transcript in different tissues is due to the lack of the endogenous exon 1, in which the intron sequencing works as an alternative 5′UTR [9]. The stability of this new transcript in comparison to the *Zfp36l2* WT transcript does not seem to be different [5]. Interestingly, ovaries from homozygous *ΔN-Zfp36l2* females express this new transcript at levels 70% lower than the levels observed in WT animals [9]. Female mice of the C57BL/6NTac strain that are homozygous for this mutant gene, *ΔN-Zfp36l2,* ovulate, and their ova can be fertilized *in vivo,* but their embryos do not progress beyond the two-cell stage of development [9].

Intriguingly, we observed that when *ΔN-Zfp36l2* C57BL/6NTac females were subjected to superovulation protocols they released only 60% as many eggs as their WT littermates (Table 1), despite evidence of normal ovarian histology [9]. Based on this observation and our previous finding of a dramatic decrease in *ΔN-Zfp36l2* transcript levels, particularly in these animals' ovaries, we hypothesized an additional defect in ovulation and/or oocyte maturation, prior to fertilization and early embryonic life. In order to test this proposal, we introduced the *ΔN-Zfp36l2* mutation into the 129S mouse strain, which has been reported to respond better to superovulation protocols [10]. Unexpectedly, homozygous females for the mutation (-/-), referred to here as, *ΔN-Zfp36l2* in the 129S6/SvEvTac strain (*ΔN-Zfp36l2* C57BL/6NTac cross to the 129S6/SvEvTac strain for three generation, F3) failed to release any oocytes using superovulation protocols, prompting us to investigate oocyte maturation *ex vivo*.

**Table 1:** Sexual behaviour and ovulation of WT and *ΔN-Zfp36l2* homozygous females in the C57BL/6NTac strain upon superovulation.

Genotype	Vaginal Plugs	Total ova	Ova/female
WT (*n* = 9) a	7	170	24
*ΔN-Zfp36l2* (*n* = 9) a	6	86	14

a Adult females (5–8 weeks-old) were subjected to exogenous hormone injections to induce superovulation, according to standard procedures [48], and then mated with WT males. The next morning, vaginal plugs were checked and *in vivo* fertilized ova were recovered from the swollen ampulla 17–19 hours after hCG injection.

The results of our experiments led us to propose that the RNA binding protein, ZFP36L2, is able to down regulate the LHR mRNA, and that in a physiological context, impaired down regulation of LHR mRNA by ZFP36L2 results in anovulation and, indirectly or directly, influences oocyte maturation. To our knowledge, this is the first time that a specific target mRNA *in vivo* is found to be imbalanced when ZFP36L2 function is compromised due to low levels of ZFP36L2 expression.

## Results

### *ΔN-Zfp36l2* females do not ovulate, even under pharmacological conditions

To test our hypothesis that ZFP36L2 could influence ovulation and/or oocyte maturation, we introduced the *ΔN-Zfp36l2* mutation, originally described in the C57BL/6NTac strain [9], into the 129S6/SvEvTac strain. Unexpectedly, females carrying both copies of the *ΔN-Zfp36l2* gene in the 129S strain (F3 backcrossing) consistently failed to release oocytes using superovulation protocols in three different experiments (Table 2). The lack of ovulation in response to superovulation protocol was observed in two independent *ΔN-Zfp36l2* crosses of C57BL/6 mouse lines (V1 and V2) into the 129S6SvEvTac background (F3), each using two independently targeted ES cell lines described previously [9].

**Table 2:** Lack of ovulation in *ΔN-Zfp36l2* homozygous females when into the 129S6SvEvTac background (F3).

Genotype	Line	Vaginal Plugs	Total ova	Ova/female
WT a	V1 (*n* = 4)	3	84	28
	V2 (*n* = 4)	4	108	27
*ΔN-Zfp36l2* a	V1 (*n* = 5)	4	0	0
	V2 (*n* = 4)	3	0	0

a The experimental design was similar to that described in Table 1, except that the F3 backcrosses were derived from two independent lines (V1 and V2) originally in C57BL/6NTac mouse.

### *ΔN-Zfp36l2* oocytes failed to mature *ex vivo*, remaining at the germinal vesicle stage

**Figure 1:**
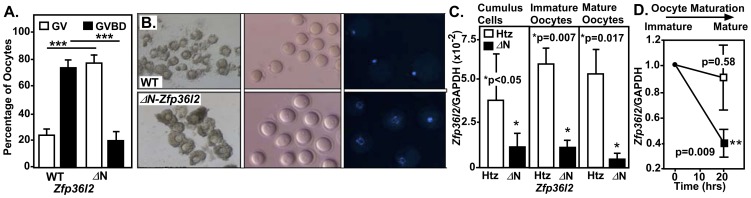
*ΔN-Zfp36l2* oocytes remain arrested at the germinal vesicle stage. Cumulus oophorus complexes (COCs) were allowed to mature *ex vivo*. After 20 hours, the oocytes were denuded by pipetting and were morphologically scored as immature (germinal vesicle, GV) or mature (germinal vesicle breakdown, GVBD) using a stereomicroscope. (**A**) Progression of oocyte maturation. Bars are means ± SD (n = 6). (**B**) Representative images of live WT and *ΔN-Zfp36l2* oocytes. *Left panel*, intact COCs (40X); *center panel,* denuded oocytes (80X); *right panel*, confocal images (40X) of *ex vivo* matured DAPI stained. (**C**) Quantification of *Zfp36l2* mRNA during oocyte maturation. Total RNA was isolated from cumulus cells, immature (GV) and mature (GVBD) oocytes from heterozygous (Htz) and *ΔN-Zfp36l2* mice after *ex vivo* maturation; *Zfp36l2* mRNA expression levels were evaluated by real-time PCR (TaqMan) and normalized to GAPDH. Bars are means ± SD of four independent biological experiments, each in duplicate. (**D**) *Zfp36l2* mRNA levels in immature oocytes were arbitrarily set as 100%, and the mRNA values in mature oocytes were calculated as percentages of the values found in immature oocytes. ‘p’ values were obtained from Student's t-test. ***p<0.001.

Given these results, we reasoned that the inability to ovulate could also be associated with an oocyte maturation problem. Therefore, we investigated oocyte maturation *ex vivo*. Since the homozygous ***Δ*** *N-Zfp36l2* females failed to ovulate, we decided to collect the oocytes surrounded by its cumulus cells, cumulus oophorus complexes (COCs), directly from the ovaries from homozygous ***Δ*** *N-Zfp36l2* young females. The oocytes surrounded by their cumulus cells were freed from the follicles and allowed to mature *ex vivo* for 20 hours. We observed that in the 129S strain (F3) 75 ± 4% of the WT oocytes matured *ex vivo*, as judged by their progression through germinal vesicle breakdown (GVBD) and the appearance of a first polar body (Fig.1A and 1B). Remarkably, only 21 ± 6% of *ΔN-Zfp36l2* oocytes matured, and the bulk (79 ± 6%) remained arrested at the germinal vesicle (GV) stage (Fig.1A and 1B). To confirm the morphological classification of the maturation stages in WT and *ΔN-Zfp36l2* oocytes, we next inspected the chromosome organization in these oocytes by DAPI staining and confocal imaging. While the majority of WT oocytes progressed to metaphase II (MII) with condensed chromosomes (Fig.1B, upper right panel), the *ΔN-Zfp36l2* oocytes were arrested at an immature stage with their chromosomes still dispersed in a prophase I pattern characteristic of the GV stage (Fig.1B, lower right panel). This observation indicated that oocytes derived from homozygous *ΔN-Zfp36l2* females have processes involved in oocyte maturation inhibited or, alternatively, have processes involved in the maintenance of meiotic arrest potentiated.

### *Zfp36l2* mRNA expression during oocyte maturation

Given that the extent of the decrease of *ΔN-Zfp36l2* mRNA in this mouse model varies according to the tissue, we quantified its expression during oocyte maturation in heterozygous and *ΔN-Zfp36l2* mice. Of note, the heterozygous females had no apparent phenotype problem. They showed normal ovulation rates and normal oocyte maturation *ex vivo*; thus, heterozygous females were used as controls to quantify the *Zfp36l2* expression. The COCs from each group were cultured *ex vivo* for 20 hours to foster oocyte maturation. The oocytes were then denuded and scored as immature or mature. Data were collected from three independent experiments and total mRNA was extracted from isolated cumulus cells, immature (GV) and mature (MII) oocytes from both groups of mice. *Zfp36l2* mRNA levels were evaluated by real-time PCR (TaqMan) and normalized to GAPDH. We observed that *Zfp36l2* transcript levels were severely reduced in both compartments, somatic and gamete cells, derived from *ΔN-Zfp36l2* mice when compared to heterozygous animals (Fig. 1C). In order to better interpret these results in the context of oocyte maturation, the *Zfp36l2* mRNA level in immature oocytes was arbitrarily set as 100% and the mRNA values in mature oocytes were expressed as percentages of the values obtained in immature oocytes. We observed that in heterozygous females there was a slight, but not significant, decrease of *Zfp36l2* mRNA expression in mature oocytes when compared to immature oocytes (Fig. 1D). However, the mature oocytes derived from *ΔN-Zfp36l2* females showed a drastic decrease of *Zfp36l2* mRNA levels when compared to its own levels in immature oocytes (Fig. 1D). This finding suggests that the *ΔN-Zfp36l2*-linked arrest is associated with low expression level of ZFP36L2 in mature oocytes.

### PKA inhibition partially rescues *ΔN-Zfp36l2* oocyte maturation

**Figure 2:**
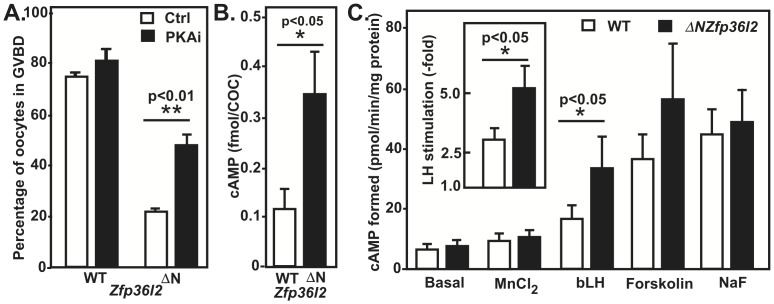
(**A**) PKA inhibition partially rescues *ΔN-Zfp36l2* oocyte maturation. WT and *ΔN-Zfp36l2* COCs were treated with 0.5 mM of Rp-cAMPS. The oocytes were denuded from the surrounding cumulus cells and classified as immature (GV) or mature (GVBD). Bars are means ± SEM (n = 5). (**B**) *ΔN-Zfp36l2* oocytes exhibit higher levels of basal cAMP. Bars are means ± SEM (n = 5 groups of 20 COC per condition). (**C**) Adenylyl cyclase (AC) activity in ovarian homogenates of WT and *ΔN-Zfp36l2* females. AC activity was determined in the absence (basal) or presence of MnCl_2_ (2 mM in place of MgCl_2_), LH (bLH 10 mg/mL), forskolin (100 mM), and NaF (10 mM). Values are mean ± SEM (n = 6, in each group). Each reagent (or no reagent, for basal condition) was incubated with the ovarian homogenate for 20 min at 37°C. AC activity is expressed in pmol cAMP formed/min/mg protein. ‘p’ values were calculated using Student's t-test.

cAMP-dependent protein kinase (PKA) is critical for maintaining the oocyte arrested in its first meiotic prophase. *In vivo*, luteinizing hormone (LH) surge triggers a sequence of events that ultimately decrease cAMP levels in the oocyte, reducing PKA activity and allowing the oocyte to initiate an irreversible maturation process that results in its progression to a metaphase II stage [11]. To determine whether inhibiting PKA could help to overcome the meiotic arrest of *ΔN-Zfp36l2* oocytes, we treated WT and *ΔN-Zfp36l2* COCs cultured *ex vivo* with a PKA inhibitor, Rp-cAMPS. Interestingly, treatment of *ΔN-Zfp36l2* oocytes with this inhibitor doubled the percentage of oocytes able to overcome the meiotic arrest (Fig. 2A), suggesting that excessive PKA activation is a component of *ΔN-Zfp36l2*-linked arrest. The addition of the PKA inhibitor to WT oocytes had no effect in the oocyte maturation rates when the ZFP36L2 levels were normal in WT animals, as can be seen Fig. 2A.

### cAMP levels are increased in *ΔN-Zfp36l2* COCs

It is well accepted that the oocyte meiotic arrest is dependent on high concentrations of the second messenger 3′5′cyclic AMP (cAMP) [12], although the source of the cAMP is controversial. Some results suggest that the granulosa cells act as the main source of cAMP for the oocyte as cAMP may diffuse through gap junctions connecting granulosa cells to the oocyte, thereby maintaining oocyte meiotic arrest [13], [14], [15], but there are also reports suggesting that the oocyte itself can synthesize cAMP [16], [17]. Based on that information, we hypothesized that *ΔN-Zfp36l2* oocytes would exhibit high levels of cAMP. To test this, we measured cAMP levels in COCs using a sensitive technique: homogeneous time-resolved fluorescence (HTRF). As shown in Fig. 2B, *ΔN-Zfp36l2* COCs exhibit 3.5-fold higher basal cAMP levels in comparison to COCs derived from heterozygous females.

The observation that excessive PKA activation is a component of *ΔN-Zfp36l2*-linked meiotic arrest and that *ΔN-Zfp36l2* oocytes exhibit increased cAMP levels suggest that normal levels of ZFP36L2 would be necessary to decrease cAMP levels and lower PKA activity, thus allowing normal oocyte maturation. Therefore, normal levels of the RNA-binding protein ZFP36L2 play an important role in the release of the meiotic arrest imposed by cAMP.

### *ΔN-Zfp36l2* ovaries show increased LH-stimulated adenylyl cyclase activity

LH exerts its actions on ovarian tissues by stimulating its membrane receptor (LHR), which in turn activates, via Gαs-GTP, the enzyme adenylyl cyclase (AC) that catalyzes the production of the second messenger, cAMP (reviewed in [18]). Given the increased levels of cAMP observed in COCs, we studied the AC function in *ΔN-Zfp36l2* ovaries. To this end, we used ovarian homogenates from WT and *ΔN-Zfp36l2* young females. The activity of AC was accessed under various physiological and pharmacological conditions (Fig. 2C). We found a consistent and significant increase of AC activity in homogenates from *ΔN-Zfp36l2* ovaries when measured in the presence of LH, thus through LHR activation. In contrast, when AC activities stimulated by forskolin and sodium fluoride, and as seen in the presence of low concentrations of MnCl_2_ (2 mM) were measured, no difference was observed between WT and *ΔN-Zfp36l2* mice, suggesting that ZFP36L2 does not directly affect the activation of adenylyl cyclase.

### Up Regulation of LH Receptor in *ΔN-Zfp36l2* Ovaries

**Figure 3:**
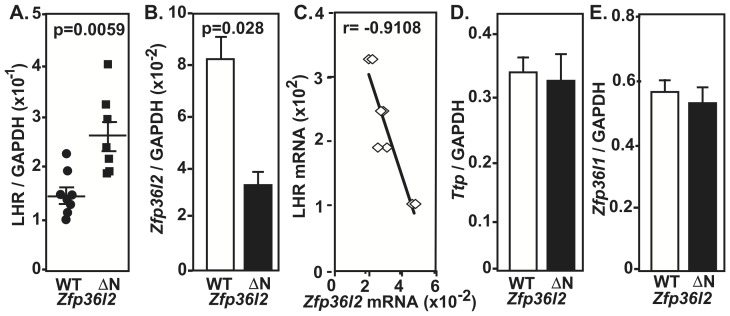
Expression of LHR and *Zfp36* family mRNAs in synchronized ovaries from WT and *ΔN-Zfp36l2* females. Total RNA was extracted from ovaries harvested on the morning on which the vaginal plug was detected. (**A**) LHR mRNA levels in ovaries from *ΔN-Zfp36l2* mice are significantly higher when compared to WT. (**B**) *Zfp36l2* mRNA was lower in *ΔN-Zfp36l* ovaries when compared to WT counterparts. (**C**) LHR and *ΔN-Zfp36l2* mRNA levels are inversely correlated, Pearson correlation coefficient  =  -0.9108. The mRNA levels of the other ZFP36 family members, *Ttp* (**D**) and *Zfp36l1* (**E**) were similar in WT and *ΔN-Zfp36l2* ovaries. ‘p’ values were calculated using Student's t-test.

Because only the LH-stimulated AC activity was increased in *ΔN-Zfp36l2* mice, we hypothesized that this may be due to increased LHR expression in these animals, prompting us to investigate the LHR mRNA levels in the ovaries. Given that the expression of the LHR is affected by the endogenous pre-ovulatory LH surge [19], we collected the ovaries in a synchronized manner after the LH surge. Total RNA was extracted from ovaries harvested on the same morning as the vaginal plug was detected (Day 0.5). As illustrated in Fig. 3A, we found a significantly higher level of LHR mRNA in ovaries from *ΔN-Zfp36l2* mice when compared to WT synchronized ovaries.

We then quantified *Zfp36l2* mRNA levels in the same samples using real-time PCR. A 65% reduction of *Zfp36l2* mRNA was seen in these cycle-synchronized ovarian samples from *ΔN-Zfp36l2* mice in comparison to WT littermates (Fig. 3B). This reduction of *Zfp36l2* mRNA in ovaries is consistent with our previous quantification in non-synchronized ovarian samples from C56BL/6 mice using Northern blots [9]. Remarkably, only in synchronized ovarian samples of *ΔN-Zfp36l2* animals, we found an inverse correlation between the levels of *Zfp36l2* mRNA and those of LHR mRNA in ovaries (Fig. 3C). Using Western bot assays we had shown that the protein levels of ZFP36L2 in ovaries reflect the mRNA levels for the transcript [9], as one would expect that the decreased levels of Zfp36l2 transcript would also be reflected at the protein level. Of note, the mRNA levels of the other members of this family of zinc finger proteins, *Ttp* and *Zfp36l1* mRNA, were similar in these ovarian samples from WT and *ΔN-Zfp36l2* animals (Fig. 3D and E, respectively).

### ZFP36L2 Binds to the LHR mRNA

**Figure 4:**
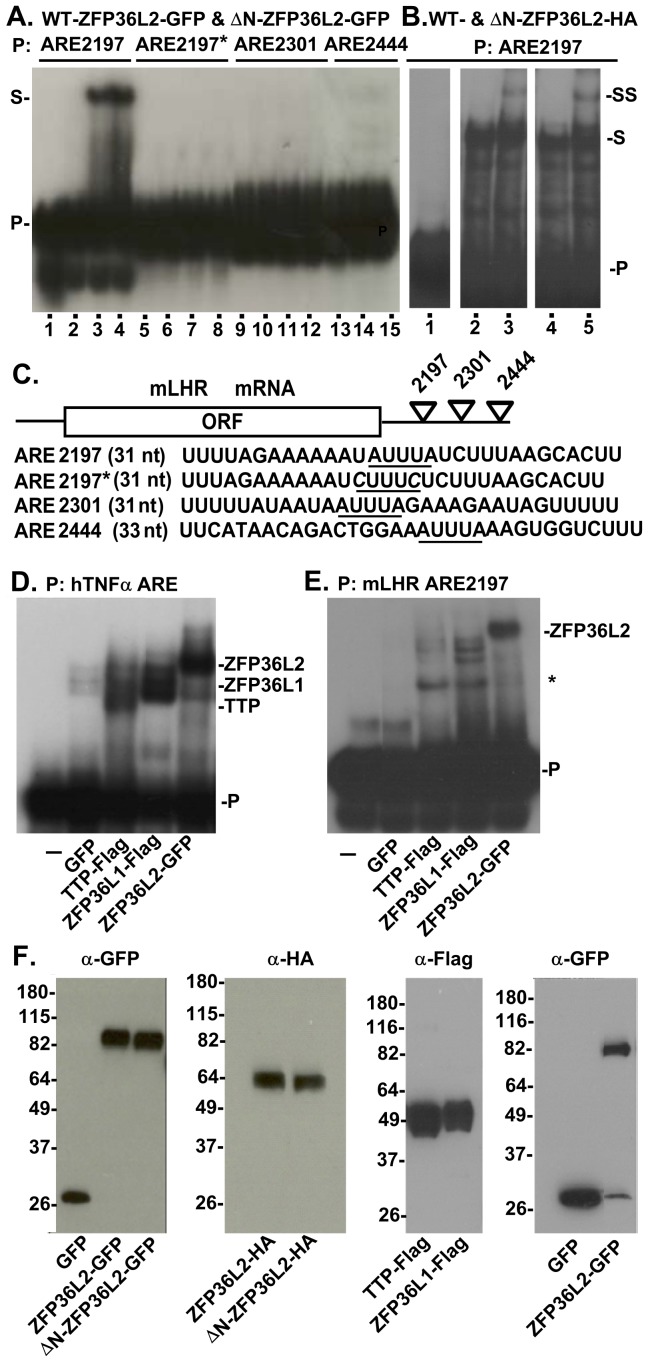
Interaction of ZFP36L2 protein with LHR mRNA. RNA electrophoretic mobility shift assays were performed by incubating protein extracts from HEK 293 cells transfected with different constructs with 2×10^5^ cpm of mLHR ARE probes. Four ^32^P-labeled mLHR probes were used as shown in panel (**C**). (**A**) Overexpressed ZFP36L2-GFP and ΔN-ZFP36L-GFP caused a shift in the electrophoretic mobility of the ARE2197 probe (S, lanes 3 and 4) with respect to the original migration of the probe (lane 1, P). GFP caused no shift (lane 2). Lanes 1, 5, 9: no protein extract; lanes 2, 6, 10 and 13, GFP controls, lanes 3, 4; 7, 8; 11, 12; 14, 15 contain protein extract from cells expressing GFP-tagged WT and ΔN-ZFP36L2, respectively. (**B**) Overexpressed ZFP36L2-HA and ΔN-ZFP36L2-HA were incubated with the mLHR probe ARE2197 in the absence (lanes 3 and 6, respectively) or presence of HA-antibody. The antibody shifted the migration of both protein-RNA complexes (supershift, SS, lane 4 and 6, respectively). The film was overexposed to clearly show the supershift. (**D**) Overexpressed hTTP-Flag, hZFP36L1-Flag and ZFP36L2-GFP caused a shift of the electrophoretic mobility of the hTNF-α ARE probe (lanes 3, 4 and 5, respectively) with respect to the probe's original migration (lane 1, P). GFP (lane 2) caused no shift. (**E**) TTP and ZFP36L1 do not seem to bind to mLHR-ARE2197 probe (lanes 3 and 4, respectively) as strongly as ZFP36L2 (lane 5). The * indicates new bands that appeared only in the presence of TTP and ZFP36L1 (lanes 3 and 4, respectively), and were not seen in the probe alone (lane 1) or in the presence of GFP (lane 2). (**F**) Western blots showing similar expression levels of proteins used in the gel shift assays mentioned above.

One mechanism by which LHR levels could be decreased after the LH surge is through its mRNA down-regulation. ZFP36L2 is an RNA-binding protein that specifically targets AU-rich elements (AREs) and leads to the deadenylation and degradation of these transcripts [4], [5]. A bioinformatic analysis identified three putative AREs in the LHR 3′UTR [http://rna.tbi.univie.ac.at/cgi-bin/AREsite.cgi], which are conserved among different species. Thus, we hypothesized that ZFP36L2 could bind to one or more of the three LHR mRNA AREs. To investigate this possibility, we performed RNA gel shift assays using three different probes, each containing one of the ARE sequence of the mouse LHR mRNA. The location of these probes at the 3′UTR of mLHR and their sequences are schematically shown in Fig. 4C. As shown in Fig. 4A, GFP-tagged ZFP36L2 and ΔN-ZFP36L2 bind to the LHR mRNA probe containing the ARE sequence located at nt 2197–2201 (lanes 3 and 4, respectively). Moreover, when both *A* residues in the heptamer U*A*UUU*A*U were mutated to *C* in ARE2197 the binding to both proteins was abolished (lanes 7 and 8), suggesting that this binding is indeed mediated by this AUUUA sequence in the 3′UTR of the LHR mRNA. Of note, no binding for either protein was observed when similar sized probes containing the ARE sequences located at nt 2301 or 2444 were used (lanes 11, 12 and 14, 15; respectively).

Next we aimed to confirm that ZFP36L2 protein was indeed present in these LHR-mRNA complexes. For this we used protein extracts of HEK 293 cells transfected with cDNAs coding for ZFP36L2 and ΔN-ZFP36L2 containing an HA tag at their carboxyl terminal ends to test if an HA antibody would super shift the migration of the band. As shown in Fig. 4B, the HA antibody super-shifted the bands containing both the WT-ZFP36L2-HA (lane 3) and the amino terminally truncated HA-tagged protein (lane 5). This super-shift demonstrates that ZFP36L2 and ΔN-ZFP36L2 are present in these RNA-protein complexes that bind to the mLHR-ARE2197 probe. Given that the other two mammalian family members, TTP and ZFP36L1, are able to bind to a TNF-α ARE probe, similar to ZFP36L2 [4], we investigated if they would also bind to mLHR-ARE2197. For this we prepared protein extracts from HEK 293 cells expressing hTTP-Flag, hZFP36L1-Flag and mZFP36L2-GFP and incubated each of them with the hTNF-α ARE probe [5] or with the mLHR ARE2197 probe. As shown in Fig. 4D, all three mammalian ZFP36 proteins bind to the hTNF-α ARE probe (lanes 3 to 5). Interestingly, we observed that TTP and ZFP36L1 (Fig. 4E, lanes 3 and 4, respectively) do not to bind to this target mRNA as strongly as the ZFP36L2 protein (lane 5). Western blots confirmed similar expression levels of the tagged proteins used in these gel shift assays (Fig. 4F).

### Overexpression of ZFP36L2 leads to decreased LHR mRNA levels in a cell line that endogenously express LHR

**Figure 5:**
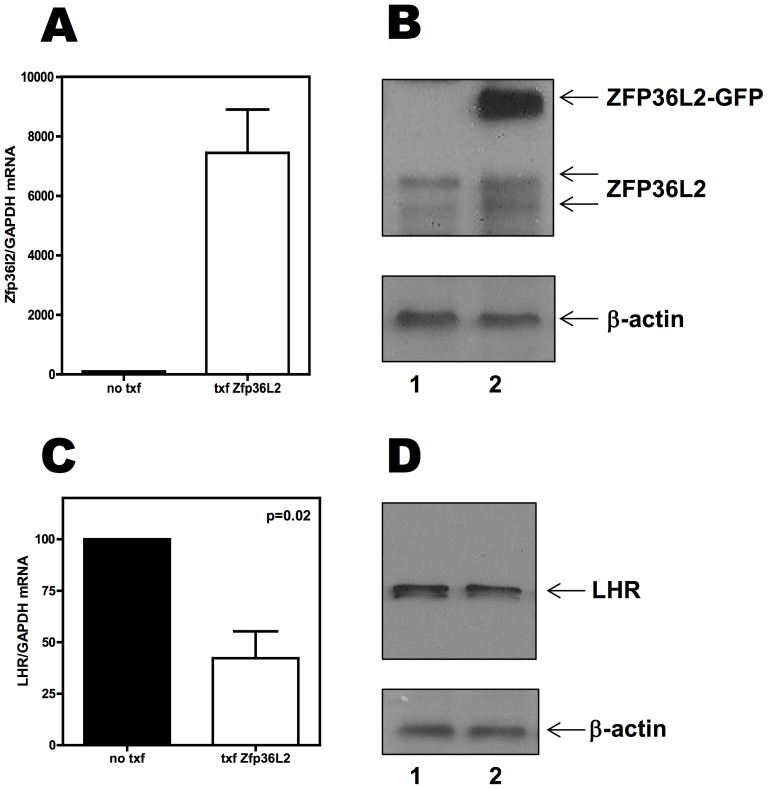
Overexpression of GFP-tagged ZFP36L2 decreases LHR mRNA endogenously expressed in MLTC-1 cells. Total RNA was extracted from MLTC-1 cells not transfected or transfected with GFP-ZFP36L2 construct. Three different sets of experiments were performed and RNA quantifications were performed in triplicate. MLTC-1 cells do express *Zfp36l2* mRNA and protein endogenously, and upon transfection the transcript levels of *Zfp36l2* were increased by 8000 fold (**A**) and an intense band of GFP-ZFP36L2 was detected (**B**). When GFP-ZFP36L2 was overexpressed; the endogenous LHR mRNA levels were 45% lower than the basal level (**C**). At the protein level, a decrease of LHR expression was also detected (**D**). ‘p’ values were calculated using Student's t-test.

Based on the gel shift assays presented above, ZFP36L2 can bind to one of the three AREs of LHR mRNA. If ZFP36L2 can bind to LHR mRNA in a physiological condition, this would result in destabilization of LHR mRNA resulting in lower levels of this transcript, excluding a purely coincidental situation. Aiming to test the effect of overexpression of ZFP36L2 in the LHR mRNA levels, we used a cell line, MLTC-1, that endogenously expresses LHR. When GFP-tagged ZFP36L2 was overexpressed in MLTC-1 cells (**Fig. 5A and B**), we detected a 40% reduction of the LHR mRNA levels, compared to the basal levels of LHR mRNA expression in these cells (**Fig. 5C**). Moreover, the decrease in LHR mRNA also results in a decrease of LHR expression (**Fig. 5D**). These experiments suggest that a causal effect of ZFP36L2 is indeed responsible for these observations.

### LH Receptor expression differs between 129S6/SvEvTac and C57BL/6NTac mouse strains

**Figure 6:**
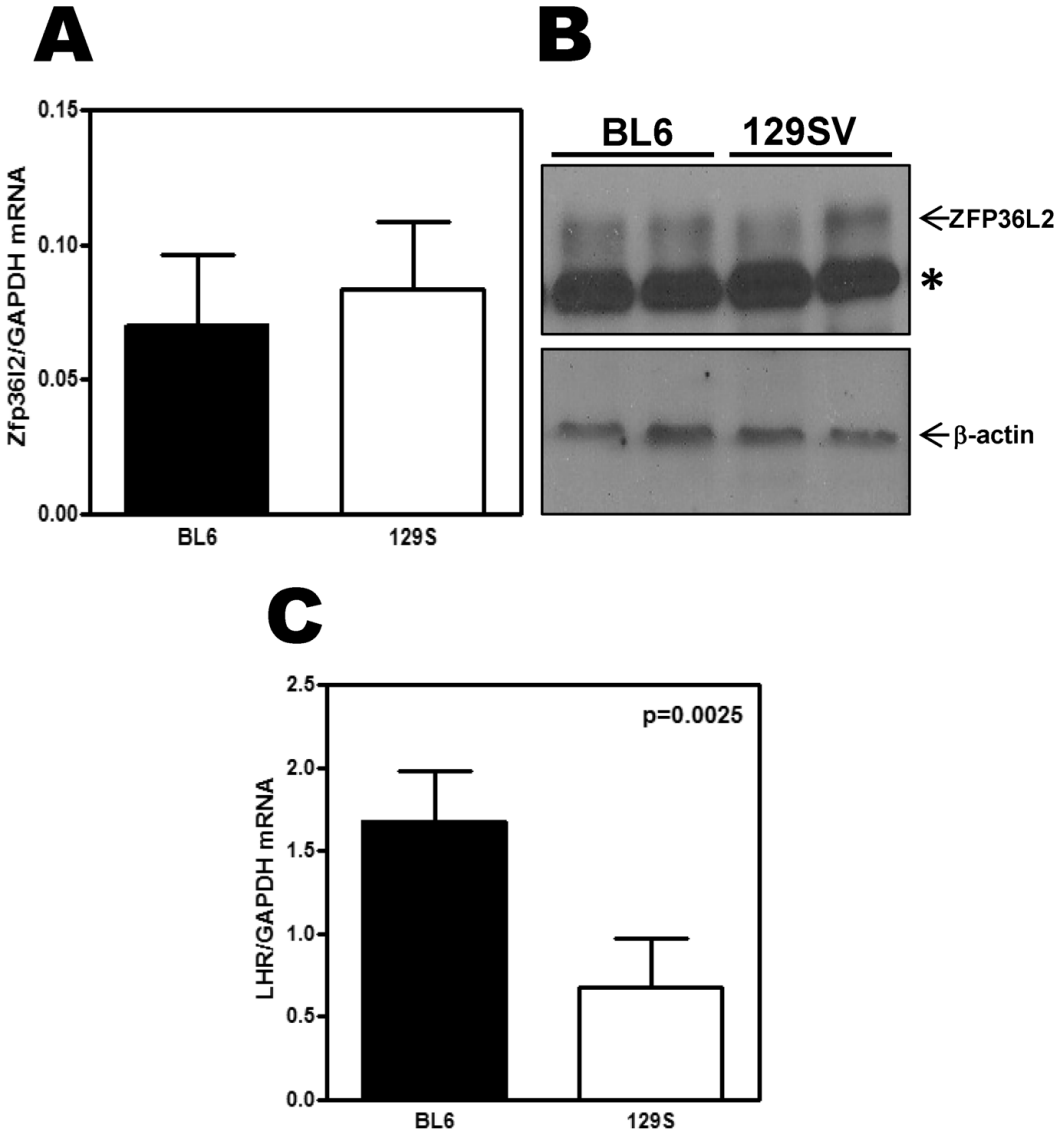
Expression of *Zfp36l2* and LHR mRNAs in synchronized ovaries from WT animals of both strains, C57BL/6NTac and 129S6/SvEvTac. Total RNA was extracted from ovaries harvested on the morning that the vaginal plug was detected from each individual female. (A) *Zfp36l2* mRNA was similar in WT animals of both strains, C57BL/6NTac (*n* = 3) and 129S6/SvEvTac (*n* = 3). (B) Western blot assays also show comparable ZFP36L2 expression in both strains, C57BL/6NTac (*n* = 2) and 129S6/SvEvTac (*n* = 2). The (*) denotes an unspecific band. (C) LHR mRNA levels in ovaries from WT 129S6/SvEvTac were significantly lower when compared to WT C57BL/6NTac. ‘p’ values were calculated using Student's t-test.

The final consequence of the *ΔN-Zfp36l2* mutation in 129S6/SvEvTac mice (F3) is the same as in C57BL/6NTac mice, complete female infertility. However, the biological basis of the infertility is the arrest of embryo development at the two-cell stage in C57BL/6NTac strain [9], while in the 129S strain (F3), is anovulation and oocyte immaturity. Aiming to investigate this difference, we compared the level of expression of ZFP36L2 and LHR in these two strains of mice. To test that we collected ovaries from C57BL/6NTac and 129S6/SvEvTac WT mice in a hormone synchronized fashion. These females were mated with stud males and every morning plugs were checked. Once the female had a positive plug, confirming that they had ovulated and had an LH surge, they were sacrificed on that morning. The levels of *Zfp36l2* mRNA and protein were comparable in both backgrounds (**Fig. 6A and B**, respectively). In contrast the levels of LHR were three fold lower in synchronized ovaries from 129S6/SvEvTac mice in comparison to C57BL/6NTac strain (**Fig. 6C**). Of note, the LHR antibody was not sensitive enough in Western blots assays to detect the endogenous protein expressed in ovaries.

## Discussion

Our data suggest that ZFP36L2 plays a key role in the paramount reproductive biological event of ovulation and influences oocyte maturation *ex vivo*, particularly in the exit from meiotic arrest imposed by cAMP. The *ΔN-Zfp36l2* mouse expresses a shorter protein, lacking the 29 amino terminal amino acids of ZFP36L2, at reduced levels when compared to its WT counterpart [9]. A comparison of the biochemical properties of the full length and truncated proteins had shown that subcellular localization of the two proteins and their ability to bind RNA and promote deadenylation is similar [5]. Thus, the *ΔN-Zfp36l2* mouse fits into the classic definition of a hypomorphic mutation in which a decreased level of the protein is the major component of the *in vivo* phenotype. Here we show that *Zfp36l2* mRNA is decreased in the ovaries of *ΔN-Zfp36l2* homozygous animals, which had unchanged levels of two other family members, *Ttp* and *Zfp36l1*. In fact, *Ttp* and *Zfp36l1* mRNA expression in ovaries were higher than that of *Zfp36l2* suggesting that they cannot compensate for ZFP36L2 function in this particular organ. These findings also reveal an important discordance between high levels of mRNA expression and actual *in vivo* function, similar to the findings of a systematic study of RNA-binding proteins expression in different tissues [20]. The low levels of *Zfp36l2* mRNA relative to the other family members may also explain the difficulty in identifying target mRNAs for this protein, particularly given the small amounts of material to which one has access when investigating oocyte maturation and early embryo development.

To our knowledge, specific mRNA targets for ZFP36L2 with functional repercussion were unknown, despite its evident role in hematopoiesis [21]. Recent experiments using deep sequencing and ChIP-seq found potential *in vivo* mRNA targets for ZFP36L2 related to erythropoiesis [22]. ZFP36L2 function has also been connected to the thymus, given that lack of both ZFP36L1 and ZFP36L2 impairs thymic development [23]. Interestingly, overexpression of individual amphibian ZFP36 proteins leads to similar consequences affecting kidney morphogenesis [24].

Using our *ΔN-Zfp36l2* mouse model, the biological events found to be compromised were ovulation and oocyte maturation, both processes known to be mediated through LHR signaling [25], [26]. For this reason, downstream signaling of the LHR was investigated, i.e. the activity of AC. Coincidentally, AC activity in *ΔN-Zfp36l2* ovaries was increased only when specifically measured in the presence of LH, but not by other agents. This led us to hypothesize that ZFP36L2 could directly modulate LHR. Initially, the LH surge does lead to a transient increase in cAMP, which triggers a dramatic down regulation of the LHR mRNA levels [27]. Not only do the LHR mRNA levels decrease in the ovaries after the LH surge and initial luteinization process [19], [27], [28], but this is also accompanied by a decrease in LH binding sites [27], [29], [30], which ultimately result in decreased levels of cAMP in granulosa cells. Thus, the LH surge triggers a mechanism by which the cAMP formed by stimulation of the LH-sensitive adenylyl cyclase culminates in LHR mRNA degradation and, consequently, loss of LHR protein.

Our gel shift assays demonstrate that overexpressed ZFP36L2 binds to a specific subset of the ARE motifs found in the LHR mRNA 3′ UTR. These experiments alone do not establish that the LHR mRNA is a physiological target; however, the fact that the other two family members, ZFP36 and ZFP36L1, do not shift this LHR ARE probe while ZFP36L2 does, suggests that this protein/RNA recognition is highly specific. In fact, not only can ZFP36L2 bind to LHR mRNA, but when ZFP36L2 is overexpressed in a cell line that endogenously expresses LHR it results in decreased levels of LHR mRNA. Thus, we propose that LHR mRNA is the first target for ZFP36L2 with physiological consequences, such as influencing ovulation and oocyte maturation. Interestingly, another protein, LHR mRNA binding protein (LRBP) [29] has also been implicated in the LHR mRNA degradation [30]. Even though its biochemical mechanism has been well studied, its biological role remains unclear.

It should be noted that the final consequence of the *ΔN-Zfp36l2* mutation in 129S6/SvEvTac (-/-) mice (F3) is the same as in C57BL/6NTac (-/-) mice, i.e. complete female infertility. Originally, we described that the biological basis of the infertility is the arrest of embryo development at the two-cell stage in C57BL/6NTac mice [9]. However, in the 129S strain (F3) despite the phenotype being the same, the biological basis, anovulation and oocyte immaturity, is more severe as it precedes two-cell stage arrest. ZFP36L2 expression levels are comparable in both mouse strains. Similarly, homozygous animals caring the *ΔN-Zfp36l2* mutation in both backgrounds express low levels of ΔN-ZFP36L2 protein, but yet the biological basis of the infertility seems to be more severe in 129S6/SvEvTac mice. Likely, part of this difference can be attributed to our observations that 129S6/SvEvTac mice have LHR mRNA levels that are three fold lower than the levels observed in the C57BL/6NTac mice. Given that the LHR mRNA pool is smaller in the ovaries of 129S6/SvEvTac mice, the decreased expression of ΔN-ZFP36L2 in this background may end up resulting in a proportional larger accumulation of LHR, resulting in a more drastic and earlier biological effect. This could potentially explain the complete inability of these females to ovulate, an event that is known to be orchestrated by LHR. Alternatively, the fact that the role of ZFP36L2 in fertility becomes apparent at different stages in the two mouse strains may suggest that ZFP36L2 could also have other mRNA targets involved in the cellular biology aside from regulating LHR mRNA levels, especially transcripts that could explain the oocyte immaturity we observed in COC from homozygous females cultured *ex vivo*. Curiously, LHRs are present in theca and granulosa cells but not in cumulus cells or oocytes [28], [31]. Thus, the effects of LH on the cumulus-oocyte complex (COC) are probably mediated by paracrine factors and/or transfer of signals through gap junctions between follicular cells [32]. It is likely that LH-induced signals propagate from the mural granulosa cells to the COC to promote resumption of oocyte meiosis [33]. In mammals, when the oocyte is removed from the follicle, it undergoes spontaneous maturation [34]; when oocytes from homozygous *ΔN-Zfp36l2* females were removed from their follicles they remain arrested at the GV stage. cAMP is an important factor in maintaining this meiotic arrest [12], [13], [14], [15], [33], [35], [36], [37], [38]. High cAMP levels in the oocyte maintain the meiotic arrest [33], [39], [40]. The *ΔN-Zfp36l2* oocytes had a 3-fold increase in their cAMP levels in comparison to the WT oocytes. High cAMP levels are likely keeping PKA in its active state, maintaining the meiotic arrest and preventing oocyte maturation. Supporting this hypothesis, when the PKA pathway was inhibited in our model, approximately 50% of the *ΔN-Zfp36l2* oocytes left the GV stage and progressed to GVBD. Interestingly, the fall in cAMP in the oocyte occurs shortly after the ovulatory LH surge. This seems paradoxical because LHR signaling increases cAMP levels in granulosa cells [41], [42]. However, the LH surge also leads to a dramatic down regulation of the LHR mRNA levels [27], which ultimately leads to decreased levels of cAMP. As of now, it is unclear if the arrest of the oocytes in GV stage we observed is dependent on LHR signaling or is related to another mRNA target of ZFP36L2. Thus, a broadened approach comparing the ovarian gene expression profile between these two genetic backgrounds could be of interest to detect not only new targets for this RNA binding protein, but also to unravel subtle differences in the signal pathways between these two mouse strains.

**Figure 7:**
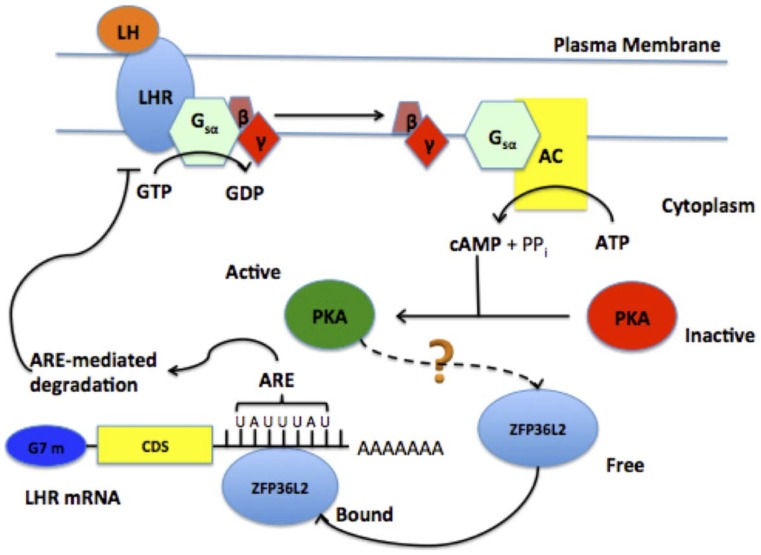
Proposed model of LH negative feedback involving ZFP36L2 RNA-binding protein. The binding of LH to LHR activates the cAMP/PKA pathway, which through an unidentified mechanism favors ZFP36L2 binding to LHR mRNA. ZFP36L2 binds specifically to one ARE, located at the 3′UTR of LHR mRNA, promoting degradation of this transcript; thus generating a negative feedback response to LH response.

In conclusion, based on the data presented here we propose that the LH surge, directly or indirectly, favors ZPF36L2 to bind the LHR mRNA at its 3′UTR, which ultimately leads to the degradation of the LHR transcript, resulting in decreased expression of LHR in the granulosa cells during the ovulation process (Fig. 7). We speculate that the low levels of ZFP36L2 (70% decrease in the ovary) in *ΔN-Zfp36l2* females are insufficient to properly down-regulate the LHR mRNA induced by the LH surge, thus resulting in anovulation.

The *ΔN-Zfp36l2* mouse allowed us to observe that low expression levels of ZFP36L2 in the ovary resulted in sustained high levels of LHR mRNA that ultimately impair ovulation and affects oocyte maturation *ex vivo*. Our data support an important role for this new specific RNA/protein interaction in the physiopathology of female infertility, pointing to ZFP36L2, as a critical factor in post-transcriptional regulation of LHR mRNA. This novel biological role for the ZFP36L2 protein in ovulation and oocyte maturation suggests a relevant function for ZFP36L2 in female reproductive disease. Possibly, mutations or genetic polymorphisms of *ZFP36L2* might be found in women with infertility presenting with anovulation and/or oocyte immaturity in IVF cycles.

## Materials and Methods

C57BL/6NTac mice carrying the *ΔN-Zfp36l2* mutation of ZFP36L2 RNA-binding protein have been described previously [9], and were housed in the vivarium of UNC's School of Medicine, Chapel Hill. All experiments were in accordance with approval of institutional animal care and use protocols according to National Institutes of Health guidelines (NIH publication 86-23, 1985). The University of North Carolina at Chapel Hill Institutional Animal Care and Use Committee (IACUC) revised and approved the animal protocols under the ID#08-201.0; either WT littermate or WT mice matched for age were used as controls.

### Isolation and Culture of Cumulus Oophorus Cells (COC)

WT and *ΔN-Zfp36l2* (-/-) 129S6/SvEvTac (F3) mice were maintained and studied in accordance with protocols approved by animal care and use committees at UNC-Chapel Hill. They were maintained on a 12 h light: 12 h dark cycle with access to food and water available *ad libitum*. Ovaries were collected from pre-pubertal (21 to 26 days old) WT or *ΔN-Zfp36l2* homozygous 129S6/SvEvTac females and placed into culture medium where they were extracted from the bursa, cleaned of adhering fat and minced. Antral follicles were punctured with a sterile needle, and full-grown oocytes surrounded by cumulus cells (cumulus oophorus complex or COC) were sorted and placed in M199 medium at 37°C, as described in details in [43], [44]. The COCs were then transferred to multi-well dishes containing 0.5 mL Waymouth medium with 5% fetal calf serum and cultured at 37°C in an atmosphere of 5% CO_2_ with 100% humidity. After 20 hours, the cumulus cells were removed by repeated pipetting, and denuded oocytes were morphologically evaluated using a stereomicroscope (60x) for spontaneous maturation *ex vivo*. Oocytes showing clear nuclear membrane and nucleoli were classified as germinal vesicle (GV) stage. Resumption of meiosis was assessed by the absence of visible nuclear structure, which was classified as germinal vesicle breakdown (GVBD). For some experiments, polar body formation, indicating progression to MII, was also scored.

### Chemical Treatment

In some experiments, drugs were added to the COC cultures, such as Rp-cAMPS (Phoenix Pharmaceuticals, CA, USA), a PKA inhibitor that binds to the regulatory subunit of PKA. A stock solution (100x) of Rp-cAMPS was prepared in culture medium and stored in aliquots at -20°C. The working stocks were further diluted in culture medium to the final concentration of 0.5 mM of Rp-cAMPS for each experiment.

### Microscopy

After *ex vivo* maturation, live oocytes were photographed directly from the stereomicroscope using a Nikon digital camera. For DAPI staining, denuded oocytes were fixed in 4% (v/v) paraformaldehyde in phosphate-buffered saline (PBS; pH 7.4) for 15 min at room temperature and then stained with 800 nM Hoechst 33258 dye for an additional 15 min. Oocytes were extensively washed and the slides were mounted using Prolong-Gold (Molecular Probes, NY, USA). Observation was performed on a Zeiss LSM 510 confocal microscope (Zeiss, Thornwood, NY, USA).

### cAMP measurement

COCs were harvested simultaneously from Htz and *ΔN-Zfp36l2* females and then incubated *ex vivo* for 30 min in M199 medium at 37°C, then snap frozen in dry ice and stored at −80°C. The same number of COCs (n = 20) per experiment from each group was collected. The concentration of cAMP was measured by homogeneous time-resolved fluorescence (HTRF) using the cAMP fento kit (CIS Bio International, Fr) and a TECAN Genios Pro instrument (Switzerland).

### Preparation of Ovarian Homogenates and Adenylyl Cyclase Assays

Ovaries were collected from young WT and *ΔN-Zfp36l2* littermate females. Ovaries from each female were individually dissected from fat in a cold PBS solution and then both ovaries from the same animal were homogenized together using a mini-polytron in 10 volumes of an ice cold medium containing 27% (wt/wt) sucrose, 1 mM EDTA, and 10 mM Tris-HCl, pH 7.5. The homogenate was then filtered through a silk screen, and 10 µl aliquots were freshly used to assay basal and stimulated adenylyl cyclase (AC) activities.

Adenylyl cyclase activity was determined by incubating 10 µL aliquots of ovarian homogenate at 32°C for 20 min in a final volume of 50 µL, which in addition had 5 µL of 250 mM Tris-HCl pH 8.0, 10 mM EDTA, 20 mM MgCl_2_, 10 mM [^3^H]cAMP (ca.15,000 cpm/assay), 10 µL 0.5 mM [α^32^P]ATP (ca.10^7^ cpm/assay), 5 µL of a nucleoside triphosphate regenerating system (RS) composed of 200 mM creatinine phosphate, 26 U/ml creatinine kinase, 25 U/ml myokinase, and 20 µL of H_2_O (basal AC activity) or other additives: 250 µM forskolin diluted in 0.5% ethanol and 0.1% bovine serum albumin (BSA), 25 mM NaF, 25 µg/mL bovine LH (diluted from a 1 mg/ml stock solution in 100 mM NaCl and 0.1% BSA) or 5 mM MnCl_2_. When MnCl_2_ was used, MgCl_2_ was omitted from the assay. The reactions were stopped by addition of 100 µL a solution containing 40 mM ATP, 10 mM cAMP and 1% SDS, pH 7.5. The [^32^P]cAMP formed was quantified by a modification [45] of the original method [46]. The results were expressed as pmol of cAMP formed per minute per mg of homogenate protein. Protein concentration in each homogenate was measured using a Bradford protein assay with BSA as standard.

### Reverse Transcriptase (RT)-PCR and Real Time RT-PCR

A total of 1 µg of RNA was used to synthesize first strand cDNA using the High Capacity cDNA archive kit (Applied Biosystems) according to manufacturer's instructions. To quantify *Ttp*, *Zfp36l1*, *Zfp36l2* and LHR mRNAs, 100 ng of first strand cDNA was combined with predesigned primer/probe sets and TaqMan Universal PCR Master Mix (Applied Biosystems). For the housekeeping gene, 8 ng of first strand cDNA was used to quantify GAPDH. All reactions were performed in triplicate in 96-well plates. Real time assays were run on an ABI 7000 sequence detector system (Applied Biosystems).

### Cell Culture, Transfections, and Protein Extracts

HEK 293 cells (American Type Culture Collection) were maintained in minimum essential medium supplemented with 10% fetal bovine serum, penicillin (100 units/mL) and streptomycin (100 µg/mL). Transient transfections of 2×10^6^ cells with expression vectors directing the synthesis of GFP, hTTP-Flag, hZFP36L1-Flag, mZFP36L2-GFP, mΔN-ZFP36L2-GFP, mZFP36L2-HA and mΔN-ZFP36L2-HA, were performed according to the manufacturer's protocol using Lipofectamine2000 (Life Technologies). The transfection mixture was incubated with the cells for 20 h and then removed; after a further 24 h incubation period with medium, cells were lysed for protein extraction.

MLTC-1 cells (American Type Culture Collection) were maintained in RPMI medium supplemented with 10% fetal bovine serum, penicillin (100 units/mL) and streptomycin (100 µg/mL). The transfection protocol was the same as for the HEK 293 cells.

### RNA Electrophoretic Mobility Shift Assay

Protein extracts from HEK 293 cells transfected with constructs driven by the CMV promoter followed by each of the ZFP36 proteins tagged with a GFP or Flag were prepared. The vector alone is referred to as GFP and the tagged constructs as mZFP36L2-GFP and mΔN-ZFP36L2-GFP; hTTP-Flag and hZFP36L1-Flag. Protein extracts were incubated with 2×10^5^ cpm of ^32^-P labeled mLHR ARE RNA probe for 15 min at room temperature in a final volume of 20 µL in a lysis buffer containing 10 mM HEPES (pH 7.6), 40 mM KCl, 3 mM MgCl_2_, 0.5 µg/µL heparin, and 1.2 µg yeast tRNA, as previously described [4] except that no RNase T1 was added. The resultant reaction mixture of protein-RNA complexes was then loaded onto 6% non-denaturing acrylamide (37.5∶1) gels and subjected to electrophoresis at 150 V for 90 min in 0.4x Tris borate/EDTA buffer. For the super-shift assays, an HA antibody (Y-11, Santa Cruz) was pre-incubated with the protein extracts for 10 min, before adding the probe. In addition, for this assay further than the protein extracts from HEK cells expressing HA-tagged ZFP36L2 and ΔN-ZFP36L2, we also used a protein extract from HEK cells not containing an HA-tagged protein.

### Preparation of RNA Probes

The RNA probes, each containing one ARE sequence of the mouse LHR mRNA, were constructed with a T7 RNA polymerase system using templates of synthetic DNA linked to a T7 promoter sequence as described [47]. The three probes encoding sequences located at the 3′UTR of mLHR mRNA were designated ARE2197, ARE2301 and ARE2444 based on the location of the AREs in the transcript ENSMUST00000024916. A fourth probe, in which the two-adenine residues of the ARE2197 were mutated to cysteine, was termed ARE2197*. The hTNF-α probe described previously [5] was used as a positive control. The RNA probes were transcribed using the Riboprobe (Promega) *in vitro* transcription system in the presence of [α-^32^P]CTP (800 Ci/mmol). The synthesized RNA probes were separated from the free nucleotides using Sephadex G25 columns and then gel purified (Fig. S1).

### Western Blot Analysis

A GFP primary antibody (JL-8, Clontech) and an anti-HA (Y-11, Santa Cruz) were diluted to a 1∶10,000 to detect overexpressed protein samples. An anti-DDK (Origene) was used at a 1∶4,000 dilution to detect overexpressed hTTP-Flag and hZfp36l1-Flag. The membranes were incubated with a secondary antibody, goat anti-mouse IgG (Santa Cruz) or goat anti-rabbit (Bio-Rad), at a 1∶10,000 dilution, both antibodies were HRP-conjugated. To detect the endogenous ZFP36L2 protein, the anti-ZFP36L2 polyclonal antibody (C2-ZFP36L2-AS, described in [5]) was used at a 1∶10,000 dilution followed by a secondary goat anti-rabbit at a 1∶10,000 dilution. As a loading control, a β-actin rabbit polyclonal antibody (N-21, Santa Cruz) was used at a 1∶5,000 dilution, followed by a secondary goat anti-rabbit at a 1∶10,000 dilution. The LHR antibody was used at a 1∶2,000 dilution (ProteinTech), followed by a secondary goat anti-rabbit at a 1∶10,000 dilution. All antibody signals were developed using a SuperSignal West Pico Chemiluminescent Substrate (Pierce) according to the manufacturer's instructions.

## Supporting Information

Figure S1 **Purification of the ^32^P-labeled RNA probes from the gel.** ^32^P-labeled mLHR and hTNF-α RNA probes were electrophoresed on a 16% polyacrylamide denaturing urea gel. The gel was exposed to a film to identify the band corresponding to the expected molecular size of each probe. Each band was excised from the gel and subsequently eluted overnight using a gel elution buffer composed of 20 mM TRIS, 1 mM EDTA, 250 mM Sodium Acetate, 0.25% w/v SDS. This guaranteed the elimination of unspecific bands such as stalled labeling products, free nucleotide and the T7 enzyme. The major product of the *in vitro* transcription radiolabeling reaction was the full length of the designed DNA templates. Occasionally, a larger product would appear as a result of T7 RNA polymerase adding one to a few more nucleotides to the -3′ end of an RNA probe during transcription. Such a case was observed during the purification of the TNF-α RNA probe (lane 2). In order to maximize the amount of product for use in downstream gel shift assays, the major product (lower band) and extended product (upper band) were purified together for the TNF probe. However, the ARE2197 LHR probe (lane 1) migrated as a single band, which was thus, excised and purified.
(TIFF)
