# Supplementary figures and images for: The RNA-Binding Protein, ZFP36L2, Influences Ovulation and Oocyte Maturation
Source: PLoS One. 2014 May 15;9(5):e97324. doi: 10.1371/journal.pone.0097324 (PMC4022657; doi:10.1371/journal.pone.0097324)

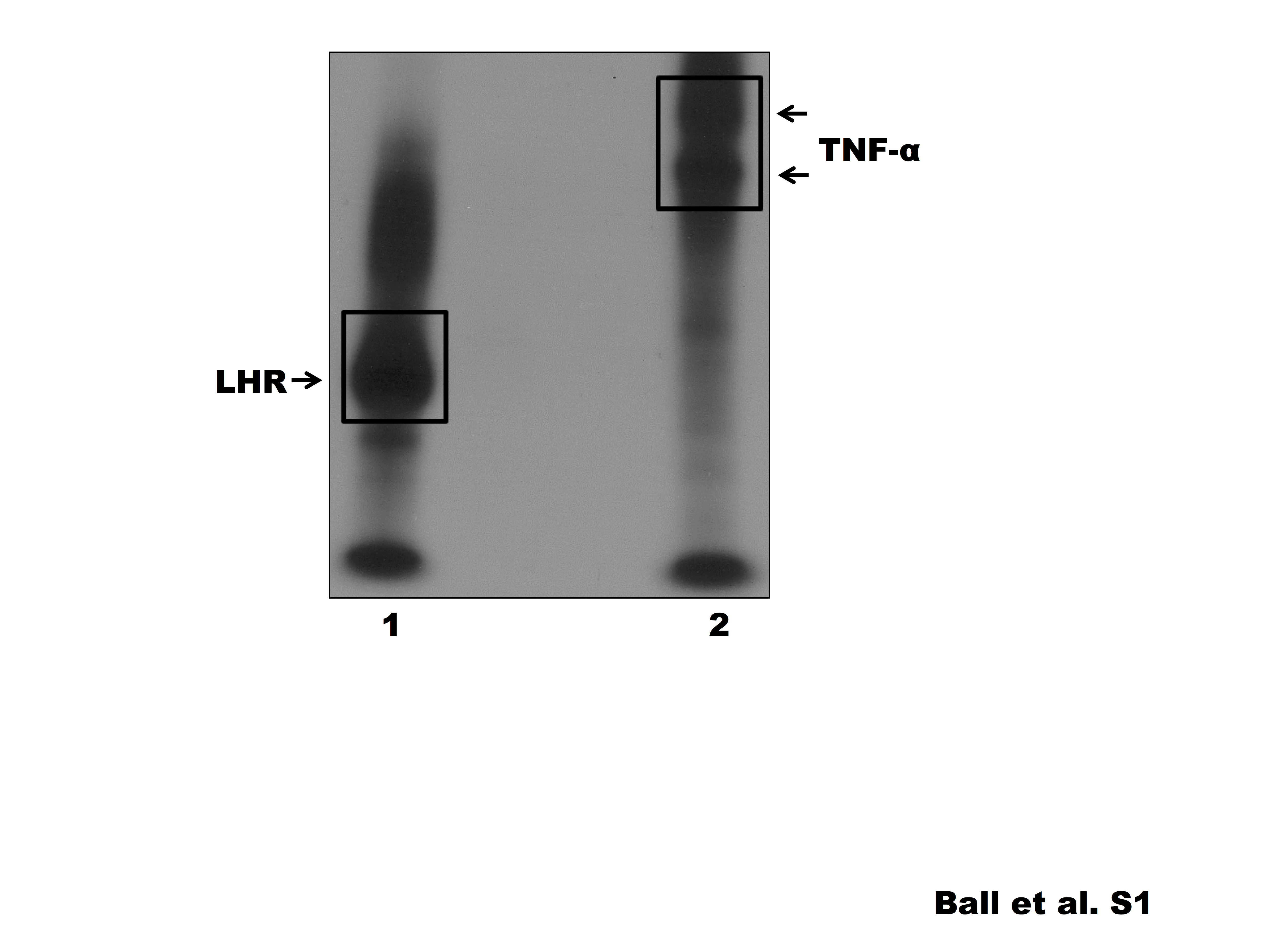

Supplement: Figure S1 — Purification of the 32P-labeled RNA probes from the gel. 32P-labeled mLHR and hTNF-α RNA probes were electrophoresed on a 16% polyacrylamide denaturing urea gel. The gel was exposed to a film to identify the band corresponding to the expected molecular size of each probe. Each band was excised from the gel and subsequently eluted overnight using a gel elution buffer composed of 20 mM TRIS, 1 mM EDTA, 250 mM Sodium Acetate, 0.25% w/v SDS. This guaranteed the elimination of unspecific bands such as stalled labeling products, free nucleotide and the T7 enzyme. The major product of the in vitro transcription radiolabeling reaction was the full length of the designed DNA templates. Occasionally, a larger product would appear as a result of T7 RNA polymerase adding one to a few more nucleotides to the -3′ end of an RNA probe during transcription. Such a case was observed during the purification of the TNF-α RNA probe (lane 2). In order to maximize the amount of product for use in downstream gel shift assays, the major product (lower band) and extended product (upper band) were purified together for the TNF probe. However, the ARE2197 LHR probe (lane 1) migrated as a single band, which was thus, excised and purified. (TIFF) [file pone.0097324.s001.tif]
